# Defining an Analytic Framework to Evaluate Quantitative MRI Markers of Traumatic Axonal Injury: Preliminary Results in a Mouse Closed Head Injury Model

**DOI:** 10.1523/ENEURO.0164-17.2017

**Published:** 2017-09-13

**Authors:** M. Haber, E. B. Hutchinson, N. Sadeghi, W. H. Cheng, D. Namjoshi, P. Cripton, M. O. Irfanoglu, C. Wellington, R. Diaz-Arrastia, C. Pierpaoli

**Affiliations:** 1 Department of Neurology, University of Pennsylvania, Philadelphia, PA; 2 Quantitative Medical Imaging Section, National Institute of Biomedical Imaging and Bioengineering, National Institutes of Health, Bethesda, MD; 3 Section on Quantitative Imaging and Tissue Sciences, Eunice Kennedy Shriver National Institute of Child Health and Human Development, National Institutes of Health, Bethesda, MD; 4 Pathology and Laboratory Medicine, Djavad Mowafaghian Centre for Brain Health, University of British Columbia, Vancouver, British Columbia, Canada; 5 Mechanical Engineering, International Collaboration on Repair Discoveries, University of British Columbia, Vancouver, British Columbia, Canada; 6 The Henry M. Jackson Foundation for the Advancement of Military Medicine, Inc, Bethesda, MD

**Keywords:** CHIMERA, diffuse axonal injury, diffusion tensor imaging, glia, immunohistochemistry, mouse

## Abstract

Diffuse axonal injury (DAI) is a hallmark of traumatic brain injury (TBI) pathology. Recently, the Closed Head Injury Model of Engineered Rotational Acceleration (CHIMERA) was developed to generate an experimental model of DAI in a mouse. The characterization of DAI using diffusion tensor magnetic resonance imaging (MRI; diffusion tensor imaging, DTI) may provide a useful set of outcome measures for preclinical and clinical studies. The objective of this study was to identify the complex neurobiological underpinnings of DTI features following DAI using a comprehensive and quantitative evaluation of DTI and histopathology in the CHIMERA mouse model. A consistent neuroanatomical pattern of pathology in specific white matter tracts was identified across *ex vivo* DTI maps and photomicrographs of histology. These observations were confirmed by voxelwise and regional analysis of DTI maps, demonstrating reduced fractional anisotropy (FA) in distinct regions such as the optic tract. Similar regions were identified by quantitative histology and exhibited axonal damage as well as robust gliosis. Additional analysis using a machine-learning algorithm was performed to identify regions and metrics important for injury classification in a manner free from potential user bias. This analysis found that diffusion metrics were able to identify injured brains almost with the same degree of accuracy as the histology metrics. Good agreement between regions detected as abnormal by histology and MRI was also found. The findings of this work elucidate the complexity of cellular changes that give rise to imaging abnormalities and provide a comprehensive and quantitative evaluation of the relative importance of DTI and histological measures to detect brain injury.

## Visual Abstract

**Figure:**
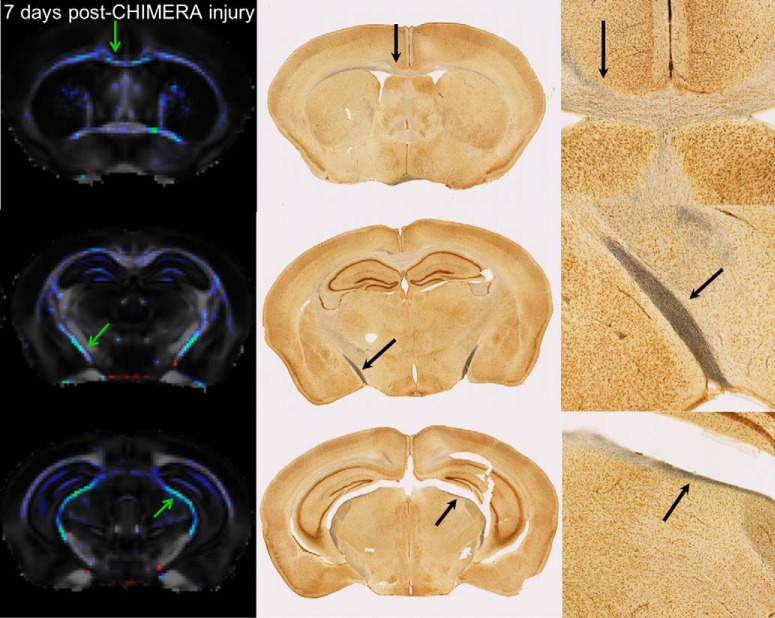

## Significance Statement

This work analyzes a recently developed brain injury model (Closed Head Impact Model of Engineered Rotational Acceleration, CHIMERA) with a clinically available imaging modality, diffusion tensor imaging (DTI), to identify pathophysiological features of traumatic brain injury (TBI) that effect diffusion imaging metrics. A comprehensive analysis of DTI metrics and histopathology was performed. Anisotropic metrics corresponded with several neurobiological features of injury including neuroinflammatory cell infiltration. A machine-learning algorithm was able to identify important regions and metrics for injury classification in an unsupervised way. The current study elucidated potential imaging biomarkers for experimental TBI as well as a statistical method for handling multiparametric data.

## Introduction

Over 2 million traumatic brain injuries (TBIs) occur in the United States every year [Center for Disease Control and Prevention (CDC), 2010]. The acute response of brain tissue to a mechanical insult to the head initiates a complex collection of secondary biological processes resulting in ongoing pathology and neurologic outcomes that appear weeks or months following the initial injury (Bramlett and Dietrich, 2015). One of the hallmark pathologies that often accompanies TBI is diffuse axonal injury (DAI), which arises acutely from the selective vulnerability of the myelinated axons to mechanical damage during TBI and results in acute and subacute disruption of axonal transport, including the appearance of retraction bulbs and axonal varicosities. In some cases, DAI can result in long-term consequences of axonal degradation and Wallerian degeneration, which are associated with compromised function (Johnson et al., 2013).

Accurate diagnosis of TBI is clinically challenging, as there is no validated means of predicting which patients will have long lasting deficits and which will recover without intervention. Advances in noninvasive imaging approaches including magnetic resonance imaging (MRI) may help to close this gap. Computed tomography (CT) and structural MRI are commonly used clinical tools for diagnosis of TBI, but fail to identify more subtle pathology (Bazarian et al., 2007; Duhaime et al., 2010; Haacke et al., 2010; Shenton et al., 2012; Riedy et al., 2016). Diffusion tensor imaging (DTI; Basser et al., 1994; Pierpaoli et al., 1996) has the unique capability to measure the diffusion of water in tissue as a proxy for microstructural and architectural properties and has shown promise for detecting TBI abnormalities in several clinical and preclinical studies (Arfanakis et al., 2002; Bazarian et al., 2007; Mac Donald et al., 2007a; Mac Donald et al., 2007b; Hulkower et al., 2013; Li et al., 2013; Yuh et al., 2014). Despite the potential for diffusion MRI methods to provide a crucial role in the diagnosis, prognosis, and experimental study of DAI, the relationships between DTI markers and their neurobiological underpinnings remain to be well established (Budde et al., 2011; Zhuo et al., 2012). Integration of DTI with preclinical studies will allow for a better understanding of diffusion imaging abnormalities that may ultimately be translated into the clinic.

The recently developed Closed Head Impact Model of Engineered Rotational Acceleration (CHIMERA) uses precise delivery of acceleration of the head to reliably create DAI in several white matter tracts throughout the brain (Namjoshi et al., 2014) that mimics DAI following human TBI (Adams et al., 1982; Adams et al., 1989; Johnson et al., 2013). Detailed whole-brain, quantitative characterization of the neuropathology induced by CHIMERA experimental TBI will allow for a better understanding of DAI and other secondary injury mechanisms in humans, providing a basis for the development of neuroregenerative therapeutics and diagnostic biomarkers.

The objective of the current study was to comprehensively evaluate MRI and histologic markers of pathology in the same brain tissue to identify key DTI abnormalities that accompany DAI and also to better understand the relationship between these DTI changes to histologic measures of specific secondary injury pathology. Brain specimens were taken 7 d following CHIMERA injury and scanned *ex vivo* using high resolution and high-quality T2-weighted MRI as well as DTI. When scanning was complete, coronal sections of the entire cerebrum and the underlying neuroanatomy were stained to identify cellular alterations after injury. These observations were confirmed quantitatively by group analysis and extended by classifier analysis to identify from the rich set of histologic and DTI values, the metrics most predictive of injury status. The results of this work extend the understanding of the neurobiological substrates underlying DTI abnormalities in white matter following TBI as well as identify potential DTI biomarkers that can distinguish injured versus noninjured tissue.

## Materials and Methods

### Fixed CHIMERA TBI mouse brains

Repetitive CHIMERA injury was performed as described in Namjoshi et al. (2014). All experiments were approved by the University of British Columbia Committee on Animal Care and are compliant with the Canadian Council of Animal Care. Male C57Bl/6 mice at four months of age were used in this study. Mice were housed in actively ventilated cages in a pathogen free barrier rodent facility with environmental enrichment on a 12/12 h light/dark cycle. Mice received the 2918 Teklad Global 18% Protein rodent diet (Harland) and autoclaved reverse osmosis water *ad libitum*. All the animals in this study were male to reduce variability in this small sample study. Future studies will take into account sex as a biological variable. Mice were housed four to a cage and cage-mates were evenly distributed into the two groups of the study, CHIMERA-injured and sham. Injury was performed initially and an identical injury occurred twenty-four hours later. Briefly, male C57BL/6 mice were anesthetized with isoflurane (induced: 4.5%; maintained: 2.5–3%) in oxygen (0.9 l/min). Mice were placed supine on the holding bed and their body strapped down with two restraints, allowing the head to move freely. A piston impacted the vertex of the head with a force of 0.5J or 0.65J. Qualitative analysis of silver stain was used to determine presence of injury. All animals included in the CHIMERA injury group showed evidence of white matter damage via silver stain (*n* = 5). Sham animals were anesthetized and strapped down on the apparatus; however, they did not receive an impact from the piston (*n* = 5). Animals were then removed from the holding bed and observed until recovery of ambulatory function. Mice were sacrificed one week after the second injury and fixed with 4% formaldehyde in PBS via transcardial perfusion. Brains remained in the skull to preserve the gross anatomy and integrity of the nerves during MRI. After a postfixation period of 2 d in formaldehyde, specimens were rehydrated in several changes of PBS for a minimum of one week before imaging. During imaging the specimens were placed in a 15mm diameter NMR tube filled with fluorinert (FC-3283, 3M), which has no hydrogen nuclei and is therefore invisible to proton MRI but reduces susceptibility variations between the brain and the space around it.

### *Ex vivo* DTI

Diffusion MRI acquisition was performed using a 14T Bruker microimaging system with a 15-mm linear radio frequency coil. Three dimensional echo planar imaging (3D-EPI) with eight segments, echo time (TE) and repetition time (TR) = 38/617 ms and 1 nex was used to acquire 152 volumes with 100-μm isotropic resolution and the diffusion-weighted imaging (DWI) sampling scheme included two low b-value shells with b = 250 and 500 s/mm^2^ and six directions and two high b-value shells with b = 1500–1700 and 3000–3800 s/mm^2^ and 32 directions. Two repetitions were acquired for each DWI having opposite phase-encode directions for geometric distortion correction using DRBUDDI (Irfanoglu et al., 2015). A T2-weighted reference image was acquired using a multislice multiecho (MSME) pulse sequence with the same spatial dimensions as the DWIs and TE/TR = 30/3000 ms, nex = 1.

### Image processing

First the T2-weighted structural image was rigidly registered to template space (Johnson et al., 2010) using landmark based registration in mipav software (version 5.1.0, http://mipav.cit.nih.gov), and this aligned structural image was used as a target for registration and artifact correction of the DWIs. DWIs were processed using the TORTOISE software package (Pierpaoli et al., 2010). To correct for apparent motion due to frequency drift and the effects of diffusion gradients, all DWIs were rigidly registered to a reference DWI image with the lowest b-value and geometric distortions were corrected using the DRBUDDI algorithm to warp and combine DWIs with opposite phase encode direction (Irfanoglu et al., 2015). Corrected DWIs were fit to the DTI model using nonlinear tensor fitting and DTI index maps were calculated including the three eigenvalues (λ_1_, λ_2_, and λ_3_), fractional anisotropy (FA) and the trace (T) of the diffusion tensor (Basser and Pierpaoli, 1996). Radial diffusivity (RD) and axial diffusivity (AD) maps were computed by AD = λ_1_ and RD = (λ_2_+ λ_3_)/2. Measures of linear (prolate, “cigar-shaped” displacement) and planar (oblate, “pancake-shaped” displacement) anisotropy were computed according to Westin et al. (2002). We refer to these metrics as Westin’s linear anisotropy (WL) and Westin’s planar anisotropy (WP).

### Voxelwise DTI analysis

To compare DTI values across the whole brain for the bias-free identification of quantitative group differences, individual DTI maps for each brain were warped into a common space using a recently developed diffeomorphic and tensor based image registration technique (Irfanoglu et al., 2016). Difference maps for FA were generated to identify regions of abnormal FA by averaging the diffusion tensor volumes within each group and calculating the FA for each group, then subtracting the average FA map of the sham group from the CHIMERA-injured group. To visualize the spatial distribution of abnormalities, the difference maps were displayed as color overlay maps on the average sham DTI map where warm colors are positive values indicating elevated FA values in the CHIMERA group and cooler colors indicate negative FA values or decreased values in the CHIMERA group.

### Regions of interest (ROIs)

ROIs were selected based on regions exhibiting an abundance of silver stain in CHIMERA-injured tissue (brachium of the superior colliculus, cingulum bundle, corpus callosum, and optic tract). Several other ROIs exhibiting absence of silver stain from both white and gray matter were also investigated to use the availability of an internal negative control for each sample (external capsule, internal capsule, cortex, hippocampus, and thalamus.) Bilateral white (brachium of the superior colliculus, cingulum bundle, external capsule, internal capsule, and optic tract) and gray (cortex, hippocampus, thalamus) matter regions were analyzed along with the anterior commissure, anterior corpus callosum (bregma 0.14 mm) and posterior corpus callosum (bregma −1.82 mm). 3D ROIs for DTI metrics were manually drawn based on anatomic landmarks using MIPAV by a researcher familiar with mouse brain neuroanatomy (M.H.). Blinding to the sample’s study group was not performed in this study. Each ROI was drawn on five sequential slices (500 μm) of the rigidly aligned DTI maps (i.e., before registration for voxelwise analysis) to generate the complete ROI. The manually drawn regions were then converted into binary masks. DTI parameter values for each ROI were obtained by importing the 3D ROI binary masks generated by mipav into TORTOISE and exporting TORTOISE parameter values from the DTI maps.

### Histology

Following MRI, brains were rinsed in PBS while still in the skull and left for 24 hours at 4°C in PBS. The brains were then removed from the skull and cryoprotected using 20% sucrose in PBS then frozen for sectioning. Coronal sections were prepared from the entire cerebrum. Degenerating neurons were visualized using FD Neurosilver kit (FD Neurotechnologies) on 40-μm sections. Immunohistochemical and immunoflorescent stains were performed on 30-μm sections. Axonal damage was detected via anti-amyloid precursor protein (APP; 1:2000; catalog 51-2700; Invitrogen) and anti-neurofilament (NF; 1:300; catalog NA 1297; Biomol). Myelin sheaths were identified using an antibody against myelin basic protein (MBP; 1:3000; catalog 40390; Abcam). Neuroinflammatory cells were detected using antibodies against glial fibrillary acidic protein (GFAP; 1:30,000; catalog 13-0300; Invitrogen) and ionized calcium-binding adaptor molecule 1 (IBA-1; 1:6000; catalog 019-19741; Wako Chemicals USA) for astrocytes and microglia, respectively (Table 1). Antibody-antigen complexes were detected using appropriate peroxidase-conjugated or Alexa Fluor 488 secondary antibodies. Immunohistochemical stains used diaminobenzodine as a chromogen. All antibodies were titrated and verified by FD Neurotechnologies. Optical imaging was performed using a Nanozoomer system. Confocal florescence imaging was preformed using the Zeiss LSM 700 confocal microscopy system.

**Table 1.** Antibody information

Name	Immunogen	Specifications	Concentration
Anti-β-amyloidantibody (APP)	22 amino acid synthetic peptide derived fromthe C terminus of thehuman β-APP	Thermo Fisher Scientific; catalog 51-2700;RRID:AB_2533902;rabbit, polyclonal	1:2000
Anti-myelin basic proteinantibody (MBP)	Synthetic peptide conjugated to KLH derivedfrom within residues 150 to the C terminus ofmouse MBP	Abcam;catalog ab40390;RRID:AB_1141521;rabbit, polyclonal	1:3000
Anti-Iba1 (IBA-1)	Synthetic peptide correspondingto C terminus of Iba1	Wako;catalog 019-19741,RRID:AB_839504;rabbit, polyclonal	1:6000
GFAP monoclonal antibody(GFAP)	Enriched bovine glial filaments	Thermo Fisher Scientific;catalog 13-0300,RRID:AB_2532994;rat, monoclonal	1:30,000
Pan neurofilaments (NF)	Primate and bovine NF proteins	Enzo Life Sciencescatalog BML-NA1297RRID:AB_10539699;rabbit, polyclonal	1:300

### Immunohistochemistry analysis

Semiquantitative analysis of photomicrographs of the immunohistochemically stained sections was performed with ImageJ (Schneider et al., 2012). Sections were selected based on anatomic landmarks that corresponded with the anatomy and extent of the 500-μm ROIs analyzed for DTI maps. Jpeg images of the selected coronal sections were converted into 8-bit gray scale and then into binary images using predetermined thresholds. ROIs were drawn onto the coronal sections (M.H.). For IBA-1, GFAP, and APP, a measurement of the percentage of the area covered by immunostaining was determined by Image J software. For MBP, a measurement of the optical density of staining was acquired for each ROI. Each antibody was used to stain sections throughout the entire cerebrum with a 300-μm spacing between serial sections. To approximate the 500-μm area measured using DTI, two serial sections were analyzed per ROI and the two measurements acquired were averaged to obtain a final percentage area measurement or optical density measurement for that ROI.

### Statistical group difference tests and random forests analysis

To determine which ROIs were statistically different between groups (with *p* < 0.05), immunohistochemistry and DTI measures were analyzed separately using Graphpad Prism statistical software, version 7 (GraphPad Software). For each metric, multiple *t* tests were performed and *p* values were corrected for multiple comparisons using the Holm-Šidák multiple comparisons. Both corrected (*) and uncorrected (#) *p* values are presented.

In addition to group analysis, scatterplots were generated using the R statistical package for a single ROI to demonstrate the direct relationship between the DTI metric of FA and the four quantitative histology measures in a region of known abnormality, the left optic tract. For each scatter plot a line was chosen to represent an appropriate threshold for classification of data points into injury group based on the values of the FA or histology metric. While this representation is useful for comparing the classification ability between two metrics, correlation analysis was not performed, rather a more comprehensive machine learning classification algorithm was used to evaluate the full set of variables across DTI and histologic measures for all ROIs in a manner free from potential user-induced bias. To accomplish this, random forests, a machine-learning classifier algorithm was employed across both histologic and DTI ROI values to distinguish the most predictive metrics for classifying CHIMERA-injured and sham in an unsupervised way (Breiman, 2001). The immunohistochemistry measurements and DTI parameters from all ROIs were used as input to random forests in two separate trials, the first using the DTI metrics of: FA, T, AD, RD, WL, and WP as described previously and the second with the quantitative histology metrics: IBA-1, GFAP, MBP, and APP (using random forests package in R; Liaw and Wiener, 2002; R Core Team, 2016). R statistical package defaults for random forests were used for the analysis, except the maximum number of trees was altered to 2000. More trees were added to the analysis to insure every feature from the large selection was considered in the analysis several times. All sham-CHIMERA samples were categorized as “Sham.” All 0.65J and 0.5J-CHI samples were categorized as CHIMERA injured. A correct classification is considered classifying a sham sample as Sham and 0.65J or 0.5J-CHIMERA sample as “CHIMERA.” Incorrect classifications are sham samples being classified as CHIMERA and 0.65J or 0.5J samples being classified as Sham. Classification error was calculated based on the number of samples incorrectly classified divided by the total number of samples and reported in table form. For feature selection, we used the mean decrease in accuracy, which measures the impact of a variable on accuracy of the model. The higher this number for a given feature relative to other features the more important that feature is for classification. The mean decrease in accuracy was plotted for two ROIs, left optic tract and left brachium of the superior colliculus, for the random forests analysis of all DTI metrics and all histology parameters. No outliers were removed from any of the study analyses.

## Results

### Identification of histologic and DTI abnormalities across the whole brain

**Figure 1.**
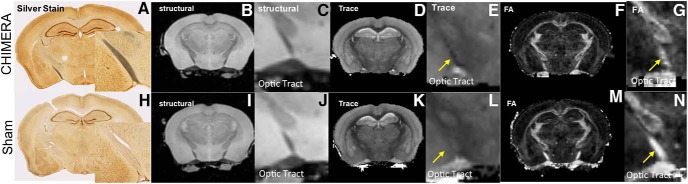
CHIMERA-injured brains exhibit DTI abnormalities in the absence of T2 abnormalities *ex vivo*. Repetitive 0.65J CHIMERA-injured brain 7 d after injury via silver stain and *ex vivo* DTI (***A–G***) compared to sham-CHIMERA fixed brain (***H–N***). The silver stain shows diffuse staining throughout the 0.65J-injured tissue, especially in the optic tract (***A***) as compared to the lack of staining in the sham (***H***). The same 0.65J and sham brains were subjected to *ex vivo* MRI imaging. Both the 0.65J (***B***, ***C***) and sham (***I***, ***J***) brains lack abnormalities in T2 imaging; however, DTI revealed differences between the injured and sham brains (***D–G***, ***K–N***). The CHIMERA-injured brain showed a reduction in T and FA in the optic tract (***E***, ***G***) compared to sham (***L***, ***N***).

**Figure 2.**
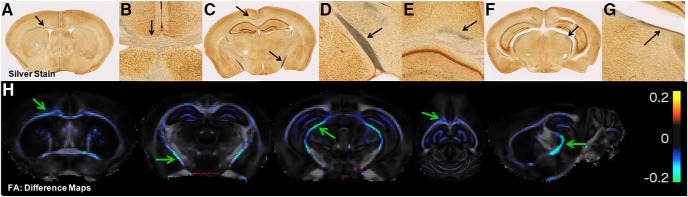
Voxelwise group differences in FA are selective for the same regions demonstrating positive silver stain. ***A–G***, Photomicrographs of silver stained sections from a 0.65J CHIMERA brain showing DAI in the anterior corpus callosum (***A***, ***B***), optic tract (***C***, ***D***), cingulum bundle (***C***, ***E***), and brachium of the superior colliculus (***F***, ***G***). ***H***, An average sham FA map (*n* = 5) was subtracted from an average CHIMERA FA map (*n* = 5) to create difference maps for FA. Colors refer to changes in anisotropy between sham and CHIMERA group averaged maps. The regions with reduced FA correspond to the regions with positive silver stain (anterior corpus callosum, optic tract, brachium of the superior colliculus, and cingulum bundle). Black arrows denote regions of positive silver stain. Green arrows denote regions of decreased FA between CHIMERA-injured group to sham group.

Silver stained histology in sections from the whole brain for each sample were visually inspected to identify brain regions with neuronal damage following CHIMERA. While no sham brains demonstrated regions of positive staining, all CHIMERA-injured brains demonstrated some degree of staining in the same set of brain regions. In particular, the optic tract was found to take up the most abundant silver stain in the CHIMERA group (Fig. 1*A*,*H*). Other regions that exhibited positive silver stain included anterior corpus callosum, optic tract, cingulum bundle, and the brachium of the superior colliculus (Fig. 2*A–G*).

Visual inspection of MRI and DTI maps was also made to identify the most salient abnormalities. T2 images of CHIMERA brains were similar to the sham controls without qualitative imaging abnormalities in the optic tract or other regions of positive silver staining (Fig. 1*B*,*C*,*I*,*J*). Quantitative DTI metrics, however, revealed visually detectable reductions of T and FA in the optic tract (Fig. 1*E*,*G*,*L*,*N*) of CHIMERA brains compared to sham.

To substantiate these qualitative observations with operator independent voxelwise analysis, FA difference maps were generated to compare the CHIMERA and sham group (Fig. 2). While no brain regions were found to have increased FA, a pattern of regions were identified with decreased FA that were similar to the silver staining pattern of abnormalities. In particular, the optic tract, brachium of the superior colliculus, and anterior corpus callosum showed reductions in FA (Fig. 2*H*). The hippocampus also exhibited a decrease in FA but there was minimal silver stain uptake in this region. Other than this exception, regions without noticeable uptake of silver stain also did not exhibit a large change in FA.

### Quantitative assessment of DTI

**Figure 3.**
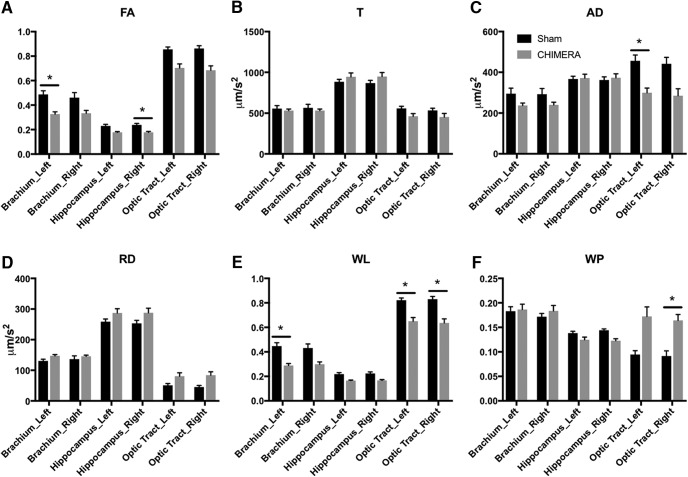
DTI measures of aniosotropy were significantly altered by CHIMERA injury. All ROIs were investigated via multiple *t* tests within a DTI parameter. After multiple comparisons correction, changes within the brachium of the superior colliculus, optic tract, and hippocampus exhibited significant differences between sham and CHIMERA-injured brains in multiple DTI metrics. ***A***, FA was significantly reduced in the left brachium of the superior colliculus and right hippocampus in CHIMERA-injured brains relative to the sham group. ***B***, CHIMERA injury did not significantly alter T in any of the regions investigated. ***C***, AD was significantly decreased in the left optic tract. ***D***, RD was altered in several regions; however, none of the changes were significant after multiple comparisons correction. ***E***, WL was significantly decreased in the left brachium of the superior colliculus as well as bilaterally in the optic tract. ***F***, WP was significantly increased in the right optic tract and decreased in the right hippocampus. The optic tract, a region which is obviously and reproducibly injured by CHIMERA, showed changes in the majority of aniosotropic DTI measures investigated. * indicates a significant *p* value after correcting for multiple comparisons (*p* < 0.05). Error bars represent the standard error of the mean.

The ROIs in the voxelwise analysis were reassessed using group analyses. DTI metrics were assessed using all 19 ROIs. Figure 3 depicts group analyses results for the bilateral brachium of the superior colliculus, hippocampus, and optic tract. Multiple *t* tests revealed changes in several ROIs (Table 2). When a multiple comparisons correction was applied, several DTI changes for FA, AD, WL, and WP anisotropy measures retained significance.

**Table 2.** Multiple independent *t* tests between results for CHIMERA-injured and sham group DTI metrics

DTI metrics	FA			T			AD			RD			WL			WP		
	*t*(8)	*p*	Adj *p*	*t*(8)	*p*	Adj *p*	*t*(8)	*p*	Adj *p*	*t*(8)	*p*	Adj *p*	*t*(8)	*p*	Adj *p*	*t*(8)	*p*	Adj *p*
Anterior commissure	1.51	0.17	0.89	1.45	0.19	0.9	1.61	0.15	0.79	0.51	0.63	0.98	1.96	0.09	0.69	1.93	0.09	0.75
Brachium_Left	4.59	***<0.01***	***0.03***	0.59	0.57	0.93	1.95	0.09	0.75	2.34	0.05	0.54	4.93	***<0.01***	***0.02***	0.24	0.81	0.99
Brachium_Right	2.71	***0.03***	0.32	0.72	0.49	0.93	1.68	0.13	0.79	0.85	0.42	0.96	3.46	***0.01***	0.11	0.93	0.38	0.96
Cingulum Bundle_Left	1.22	0.26	0.91	2.12	0.07	0.67	2.02	0.08	0.73	1.92	0.09	0.74	1.2	0.27	0.89	0.88	0.41	0.96
Cingulum Bundle_Right	0.86	0.41	0.91	1.91	0.09	0.77	1.67	0.13	0.79	2.04	0.08	0.69	0.93	0.38	0.91	0.27	0.8	0.99
Corpus Callosum_Anterior	1.42	0.19	0.91	1.1	0.31	0.92	1.84	0.1	0.77	0.37	0.72	0.98	1.34	0.22	0.89	0.31	0.76	0.99
Corpus Callosum_Posterior	1.32	0.22	0.91	2.35	***0.05***	0.6	2.11	0.07	0.7	1.74	0.12	0.75	1.69	0.13	0.91	0.67	0.52	0.97
Cortex_Left	1.41	0.2	0.91	0.58	0.58	0.93	0.73	0.49	0.93	0.45	0.66	0.98	1.26	0.24	0.89	1.47	0.18	0.91
Cortex_Right	1.2	0.27	0.91	0.41	0.69	0.93	0.55	0.6	0.93	0.31	0.77	0.98	0.89	0.4	0.91	1.44	0.19	0.91
External Capsule_Left	1.75	0.12	0.81	1.46	0.18	0.9	1.87	0.1	0.77	0.69	0.51	0.97	1.65	0.14	0.81	0.71	0.5	0.97
External Capsule_Right	0.18	0.86	0.91	2.17	0.06	0.66	1.32	0.22	0.85	2.43	0.04	0.53	1.07	0.32	0.9	1.52	0.17	0.91
Hippocampus_Left	3.77	***0.01***	0.08	1.11	0.3	0.92	0.19	0.85	0.93	1.75	0.12	0.75	3.89	***<0.01***	0.07	2.07	0.07	0.7
Hippocampus_Right	4.34	***<0.01***	***0.04***	1.35	0.22	0.9	0.44	0.67	0.93	1.92	0.09	0.74	3.88	***<0.01***	0.07	4.34	***<0.01***	***0.04***
Internal Capsule_Left	1.25	0.25	0.91	1.77	0.12	0.82	1.72	0.12	0.79	1.57	0.15	0.78	1.56	0.16	0.82	1.69	0.13	0.86
Internal Capsule_Right	0.79	0.45	0.91	1.49	0.17	0.9	1.37	0.21	0.85	1.88	0.1	0.74	0.9	0.4	0.91	1.03	0.33	0.96
Optic Tract_Left	3.9	***<0.01***	0.07	2.28	0.05	0.62	4.28	***<0.01***	***0.05***	2.43	0.04	0.53	4.82	***<0.01***	***0.02***	3.74	***0.01***	0.09
Optic Tract_Right	4.14	***<0.01***	0.05	1.53	0.16	0.9	3.39	***0.01***	0.16	3.22	0.01	0.21	4.85	***<0.01***	***0.02***	4.57	***<0.01***	***0.03***
Thalamus_Left	1.07	0.32	0.91	1.27	0.24	0.9	1.26	0.24	0.85	1.26	0.24	0.89	0.86	0.41	0.91	1.37	0.21	0.91
Thalamus_Right	1	0.35	0.91	1.09	0.31	0.92	1.11	0.3	0.85	1.07	0.32	0.93	0.67	0.52	0.91	1.43	0.19	0.91

*t* test values and *p* values are reported as well as the adjusted (Adj) *p* value after Holm-Šidák multiple comparisons.

Before multiple comparisons correction was applied, FA was decreased bilaterally in brachium of the superior colliculus, hippocampus, and optic tract (Fig. 3*A*). AD was decreased in the optic tract bilaterally (Fig. 3*C*). RD was increased in the left brachium of the superior colliculus and bilateral optic tract (Fig. 3*D*). WL was bilaterally decreased in the brachium of the superior colliculus, hippocampus and optic tract (Fig. 3*E*). WP was increased in the left and right optic tract as well as decreased in the right hippocampus (Fig. 3*F*).

After multiple comparisons correction, significant changes were seen in the left brachium of the superior colliculus for FA and WL, left optic tract for AD and WL, right optic tract for WL and WP, and right hippocampus for FA and WP (Fig. 3). Statistics for all 19 ROIs are presented in Table 2.

### Mechanistic characterization of injury in the optic tract and specific ROIs

**Figure 4.**
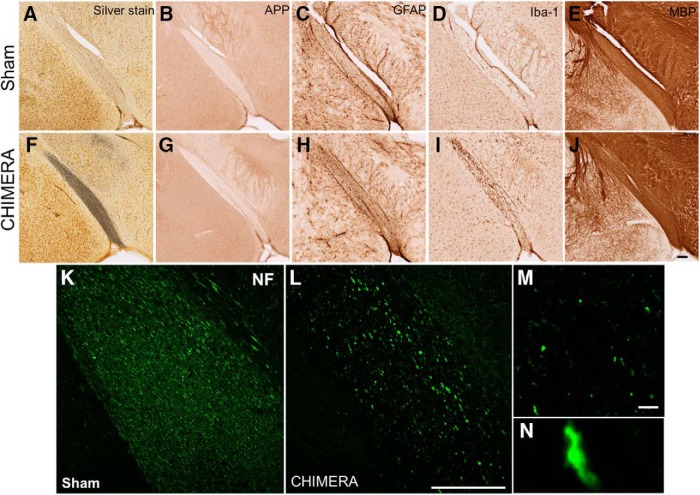
Regions with altered DTI metrics exhibit histologic abnormalities consistent with injury. Representative photomicrographs of sham (***A–E***) and 0.65J CHIMERA-injured (***F–J***) optic tract. Sections were stained with silver stain (***A***, ***F***), APP (***B***, ***G***), GFAP (***C***, ***H***), IBA-1 (***D***, ***I***), MBP (***E***, ***J***), and NF (***K***, ***L***). Scale bar = 100 μm. Retraction bulbs and axonal varicosities are prominent in magnified photomicrographs of the 0.65J tissue (***M***, ***N***). Scale bar = 10 μm.

The overlap of DTI abnormalities and positive silver staining is compelling, but while silver staining is indicative of neurodegenerating tissue, it is not informative about a specific pathophysiological mechanism. To better understand the underlying secondary injury parameters affecting the positive silver stained regions, we stained with specific antibody markers for cells commonly altered following injury (Fig. 4). APP is a commonly used marker for axonal injury. There was no change in APP immunoreactivity in the optic tract (Fig. 4*B*,*G*). Astrocyte and microglia activation were examined via increases in GFAP and IBA-1, respectively. GFAP and IBA-1 were both increased in the optic tract indicating increased gliosis and neuroinflammation (Fig. 4*C*,*D*,*H*,*I*). Myelin content was examined via MBP immunoreactivity. MBP did not show a large change between CHIMERA-injured tissue and sham brains (Fig. 4*E*,*J*). Axonal integrity was further examined using a pan-NF antibody. NF immunoreactivity revealed many retraction bulbs and axonal varicosities in the optic tract of CHIMERA-injured brains and were completely absent in the sham (Fig. 4*K*,*L*,*M*,*N*). All positive silver stained regions showed these same histologic features, however, to varying degrees depending on the severity of the injury in that region.

### Quantitative assessment of histology

**Figure 5.**
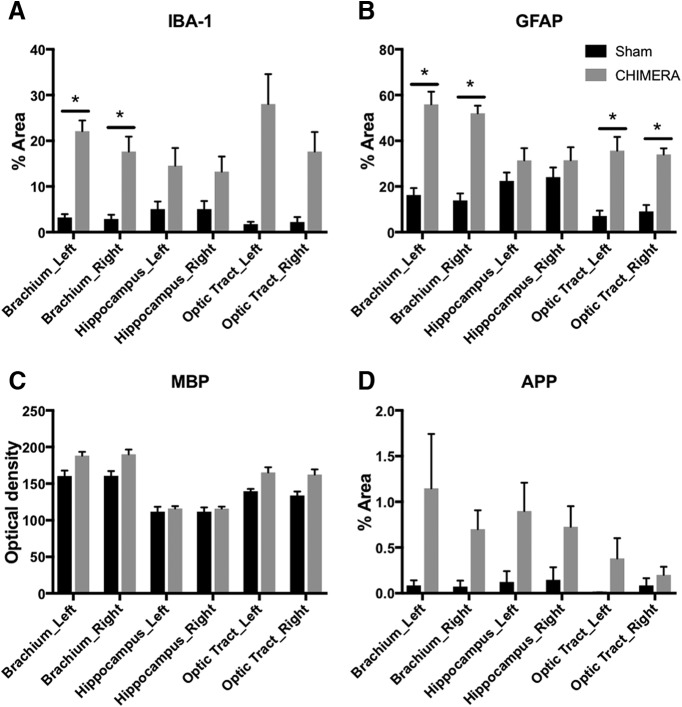
Neuroinflammation is significantly increased in white matter regions exhibiting altered DTI metrics. ***A–D***, All ROIs were investigated via multiple *t* tests within a histology marker. After multiple comparisons correction, changes within the brachium of the superior colliculus and optic tract exhibited significant differences between sham and CHIMERA-injured brains. ***A***, IBA-1 immunoreactivity is significantly elevated in the left and right brachium of the superior colliculus. ***B***, GFAP immunoreactivity is significantly elevated in the left and right brachium of the superior colliculus and optic tract. ***C***, MBP immunoreactivity is elevated in multiple regions but not significantly elevated in the brachium of the superior colliculus, hippocampus, and optic tract. ***D***, CHIMERA injury did not significantly alter APP in any of the regions investigated. The brachium of the superior colliculus and optic tract exhibit increased neuroinflammation as well as reduced FA on the difference maps. * indicates a significant *p* value after correcting for multiple comparisons (*p* < 0.05). Error bars represent the standard error of the mean.

To characterize cellular alterations across the brain, quantitative immunohistochemistry values of percentage area (IBA-1, GFAP, APP) and optical density (MBP) were analyzed for the nineteen ROIs included in this study. Figure 5 depicts group analyses results for the bilateral brachium of the superior colliculus, hippocampus, and optic tract. Multiple *t* tests revealed group differences within particular ROIs for IBA-1, GFAP, MBP, and APP (Table 3). When a multiple comparisons correction was applied, several histologic findings for IBA-1, GFAP, and MBP remained.

**Table 3.** Multiple independent *t* tests between results for CHIMERA-injured and sham group histologic markers

Histology parameters	IBA-1			GFAP			MBP			APP		
	*t*(8)	*p*	Adj *p*	*t*(8)	*p*	Adj *p*	*t*(8)	*p*	Adj *p*	*t*(8)	*p*	Adj *p*
Anterior Commissure	1.27	0.24	0.56	0.42	0.68	0.94	4.14	***<0.01***	0.06	0.84	0.43	0.67
Brachium_Left	7.7	***<0.01***	***<0.01***	6.27	***<0.01***	***<0.01***	3.11	***0.01***	0.14	1.78	0.11	0.62
Brachium_Right	4.34	***<0.01***	***0.04***	8.4	***<0.01***	***<0.01***	3.23	***0.01***	0.13	2.9	***0.02***	0.27
Cingulum Bundle_Left	1.52	0.17	0.55	2.29	0.05	0.45	2.43	***0.04***	0.16	2.8	***0.02***	0.3
Cingulum Bundle_Right	1.21	0.26	0.56	1.2	0.26	0.84	2.29	0.05	0.12	3.19	***0.01***	0.2
Corpus Callosum_Anterior	4.15	***<0.01***	0.05	3.46	***0.01***	0.12	3.45	***0.01***	0.15	1.91	0.09	0.59
sCorpus Callosum_Posterior	4.78	***<0.01***	***0.02***	2.54	***0.03***	0.39	2.71	***0.03***	0.12	1.41	0.2	0.67
Cortex_Left	3.01	***0.02***	0.17	2.33	0.05	0.45	3.45	***0.01***	***0.02***	1.28	0.24	0.67
Cortex_Right	2.34	***0.05***	0.36	1.85	0.1	0.66	5.04	***<0.01***	0.14	2.3	0.05	0.44
External Capsule_Left	3.77	***0.01***	0.07	1.55	0.16	0.79	2.99	***0.02***	0.15	1.23	0.25	0.67
External Capsule_Right	3.27	***0.01***	0.13	1.15	0.28	0.84	2.62	***0.03***	0.78	1.75	0.12	0.62
Hippocampus_Left	2.24	0.06	0.37	1.4	0.2	0.79	0.6	0.57	0.78	2.34	***0.05***	0.44
Hippocampus_Right	2.2	0.06	0.37	1.04	0.33	0.84	0.66	0.53	0.14	2.2	0.06	0.45
Internal Capsule_Left	1.6	0.15	0.55	0.54	0.6	0.94	3.16	***0.01***	0.12	2.49	***0.04***	0.41
Internal Capsule_Right	0.67	0.52	0.56	0.31	0.76	0.94	3.51	***0.01***	0.12	3.64	***0.01***	0.11
Optic Tract_Left	4.02	***<0.01***	0.06	4.42	***<0.01***	***0.03***	3.38	***0.01***	0.12	1.7	0.13	0.62
Optic Tract_Right	3.52	***0.01***	0.1	6.45	***<0.01***	***<0.01***	3.16	***0.01***	0.14	0.97	0.36	0.67
Thalamus_Left	2.69	***0.03***	0.24	2.38	***0.04***	0.45	3.87	***<0.01***	0.08	2.4	***0.04***	0.44
Thalamus_Right	1.84	0.1	0.48	1.49	0.17	0.79	3.03	***0.02***	0.14	3.91	***<0.01***	0.08

*t* test values and *p* values are reported as well as the adjusted (Adj) *p* value after Holm-Šidák multiple comparisons correction.

Before multiple comparisons correction was applied, both IBA-1 and GFAP immunoreactivity was increased in the CHIMERA group relative to sham samples for the brachium of the superior colliculus and optic tract bilaterally (Fig. 5*A*,*B*). MBP showed a slight increase in the majority of regions analyzed including the bilateral brachium of the superior colliculus and optic tract (Fig. 5*C*). APP immunoreactivity was increased in the right brachium of the superior colliculus and left hippocampus (Fig. 5*D*).

After multiple comparisons correction, significant increases were seen in the brachium of the superior colliculus for IBA-1 and GFAP and the optic tract for GFAP (Fig. 5; Table 3). Statistics for all 19 ROIs are presented in Table 3.

### Scatterplot analysis of FA and quantitative histology in the optic tract

**Figure 6.**
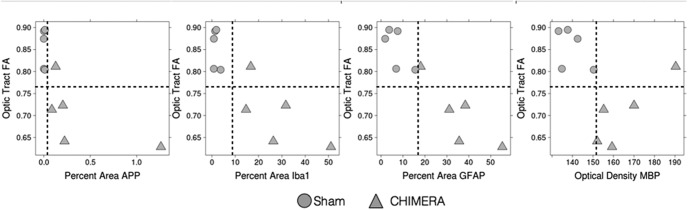
FA changes in relation to immunohistochemical alterations within the left optic tract. The optic tract is a region that exhibited very clear reductions in FA. Comparing an individual sample’s FA value to quantitative values of the immunohistochemical markers investigated revealed group wise relationships between single markers. In the left optic tract, sharp distinctions between groups can be seen based on both FA and quantitative histology values, as indicated by the dotted line. Only one injured sample is misclassified via FA values; however, all injured and sham samples can be grouped accurately via APP, IBA-1, GFAP, and MBP.

Visualization of direct relationships between DTI metrics and histopathology in a region known to be injured by several metrics, the left optic tract, is shown by the scatterplots in Figure 6. Mean FA and immunohistochemistry values are shown for the left optic tract of each brain sample included in this study. Each circle or triangle represents values from a single sham or CHIMERA brain in the study, respectively. In this region, sharp distinctions between groups can be seen based on both FA and quantitative histology values. Lines are shown that separate the data points associated with injured from uninjured samples. Using even a single metric for classification of injury status is fairly successful at distinguishing injured samples from uninjured samples as only a single point would be misclassified using FA alone. For all four immunohistological measures in the left optic tract, it is possible to classify all samples into injured and noninjured samples based on a single threshold value.

### Classification of injury via DTI and histology

**Figure 7.**
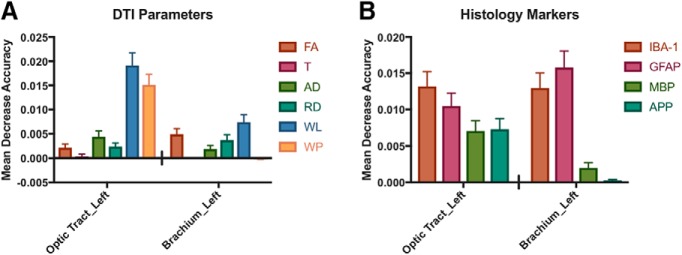
Mean decrease accuracy of DTI parameters and histology markers in specific ROIs. ***A***, ***B***, To identify metrics that contribute the most to classification accuracy for sham and injured samples, the left optic tract and left brachium of the superior colliculus were investigated. Mean decrease accuracy values and standard error were graphed for each of these regions from the random forests analysis of all DTI parameters (***A***) and all histology markers (***B***). ***A***, Classification using all DTI parameters revealed most in the left brachium of the superior colliculus and left optic tract have similar levels of importance for classification. WL and WP of the optic tract were the most informative metric; however, WP of left brachium was the least informative metric. T was not informative for classification in either region. ***B***, All histology measures of the left optic tract had high importance for the classification. IBA-1 and GFAP had the best classification ability for both regions investigated.

To extend the type of classification process demonstrated by the scatterplots in the previous section to a higher dimensional space including all DTI or histology measures across all ROIs, the random forests analysis classifier algorithm was applied and the results are summarized in Tables 4-6 and Figure 7. The analysis was performed based on two sets of features. In one case, each combination of DTI metrics and ROI is treated as a feature, and in the other, each combination of histology markers and ROI is treated as a feature. The samples were grouped into sham (*n* = 5) and CHIMERA injured (*n* = 5). Table 4 shows two combinations of parameters: (1) DTI metrics and (2) histology markers. DTI metrics or quantitative histology values from all 19 ROIs were considered during classification. Classification error was determined by number of incorrect classifications made by random forests (Table 4). When all ROIs were considered for both DTI metrics alone and histology metrics alone, 1 out of 5 sham samples was misclassified making the classification error for sham animals equal to 0.2. The same sham brain sample was incorrectly classified as CHIMERA injured by both the DTI and histology metrics. For only the CHIMERA-injured samples, all DTI metrics misclassified one out of five CHIMERA samples making the classification error 0.2. When all histology markers were considered together for only the CHIMERA-injured samples, zero out of five CHIMERA samples were misclassified exhibiting a classification error of 0. When all samples were considered together (sham, CHIMERA), overall classification accuracy of 90% was achieved using histology markers, and 80% using DTI metrics.

**Table 4.** Random forests classification accuracy for DTI and histology parameters

	Sham		CHIMERA	
	Correct	Incorrect	Correct	Incorrect
DTI parameters	4	1	4	1
*Classification error*	*0.2*		*0.2*	
Histology parameters	4	1	5	0
*Classification error*	*0.2*		*0*	

**Table 5.** Top three important features identified by random forests in classifying sham and CHIMERA-injured when using either DTI parameters or histology markers

	Top 3 ROIs	Associated metric
DTI parameters	Optic Tract_Left	WL, WP, AD, RD
	Hippocampus_Right	FA, WL, WP, RD
	Optic Tract_Right	WP, RD, FA, WL
Histology markers	Brachium_Left	GFAP, IBA-1
	Optic Tract_Left	GFAP, IBA-1
	Brachium_Right	GFAP

**Table 6.** Random forests classification accuracy for different combinations of DTI metrics

	Sham			CHIMERA		
	Correct	Incorrect		Correct	Incorrect	
FA-T	4	1		4	1	
*Classification error*	*0.2*			*0.2*		
AD-RD	4	1		4	1	
*Classification error*	*0.2*			*0.2*		
T-WL-WP	4		1	4		1
*Classification error*	*0.2*			*0.2*		

The random forests measure of mean decrease accuracy characterizes the relative importance of parameters and regions for classification. The random forests analysis feature results were ranked based on this measure and several ROIs were consistently at the top of the list. The top three ROIs for injury classification are listed alongside the DTI and histology metrics that contributed to their classification ability in Table 5. The top three regions that contributed the most to classification of sham versus CHIMERA-injured samples when using the DTI parameters were the left (WL, WP, AD, RD) and right (WP, RD, FA, WL) optic tract and the right hippocampus (FA, WL, WP, RD; Table 5). The top three regions for histology markers were the left (GFAP, IBA-1) and right (GFAP) brachium of the superior colliculus and the left optic tract (GFAP, IBA-1; Table 5).

To provide a comprehensive visual representation of the random forests analysis results, the mean decrease in accuracy is shown in Figure 7 for the two ROIs that were selected by random forests as top classifiers for each model: the left optic tract (DTI parameters) and left brachium of the superior colliculus (Table 5, histology markers). It is important to note that the mean decrease accuracy value depends on the particular parameters chosen to build the random forests. Therefore, this value may be different for a given feature depending on the parameter set of the full model and should be considered to describe the importance of a variable within the context of the chosen combination of features (Fig. 7). Classification using all DTI parameters revealed most DTI metrics of left optic tract and brachium of the superior colliculus have similar levels of importance for classification with the exception of WP of left brachium being less informative and WP of optic tract being more informative (Fig. 7*A*). TR was a poor predictor for both regions investigated.

When all histology markers were investigated, all histology measures of the left optic tract had high importance for the classification. For the left brachium of the superior colliculus, IBA-1 and GFAP are important features for classification, while MBP and APP were less important features for the classification (Fig. 7*B*).

## Discussion

### Regional abnormalities of DTI and histology

This study was designed to explore the relationship between DTI parameters and neuropathology in a DAI mouse model of TBI. The study revealed that *ex vivo* DTI abnormalities are evident following CHIMERA injury to the mouse brain in several white matter tracts even in the absence of visually detectable abnormalities in conventional structural MRI. Changes in T and reductions in FA were apparent in the optic tract, brachium of the superior colliculus, corpus callosum, and cingulum bundle. Group difference maps also revealed FA decreases in the hippocampus. When careful histologic studies were done on the same tissue, silver staining confirmed the presence of neurodegeneration in the optic tract, brachium of the superior colliculus, corpus callosum, and cingulum bundle. This pattern of silver stain reproduces the findings of Namjoshi et al. (2014) at the same time point, 7 d after injury. The colocalization of decreased FA in white matter tracts with positive silver staining suggests that FA is sensitive to the identification of DAI following closed head injury.

Beyond the identification of injured brain regions using DTI, a primary goal of this work was to better understand the cellular alterations that underlie changes in DTI metrics. To accomplish this, the regions demonstrating the greatest level of abnormality across several metrics, the optic tract, brachium and hippocampus, were carefully examined by their qualitative and quantitative ROI values for DTI and histology metrics within the same sample.

The optic tract was carefully examined in this study as it demonstrated an abundance of silver stain, as well as having the methodological advantage of high coherence and simple geometry, making it well suited for investigation by DTI. There were no gross abnormalities of the optic tract in the T2 image. Diffusion MRI, however, revealed a slight decrease in optic tract FA and RD, with a significant decreased in AD, WP, and WL anisotropy in the CHIMERA brains relative to sham. The combined decrease in FA and AD has been previously associated with axonal damage (Budde et al., 2009; Xie et al., 2010), beading (Skinner et al., 2015), and infiltrating cells (Robinson et al., 2017). Injury to the optic tract was confirmed via immunohistochemistry. In this study, APP was not altered in the optic tract and only minor evidence of APP was found qualitatively unlike reports in the white matter following CCI (Mac Donald et al., 2007b), but similar to studies of repetitive closed skull injury (Bennett et al., 2012). On the other hand, IBA-1 and GFAP were elevated and disruption of axonal integrity was found by pan-NF antibody, which demonstrated the presence of retraction bulbs and axonal varicosities throughout the optic tract. Taken together these findings are in agreement with other studies of DAI in the observation that reduced FA and AD are consistent with axonal damage, beading, infiltrating microglia and reactive glial cells. Notably, these pathophysiological mechanisms were found to be overlapping and concurrent with one another. This extends a general caveat about DTI metrics. DTI metrics are known to be sensitive to pathophysiological alterations; however, they are not specific to a particular pathophysiological mechanism. The correlation of a particular DTI metric with a specific pathophysiological mechanism does not preclude the presence of other concurrent pathology that may contribute as much or more to abnormal water diffusion.

In addition to changes in FA, AD, and RD, the optic tract had significantly reduced WL and increased WP. These metrics are less commonly used than other DTI metrics, but provide increased information about the shape of the diffusion ellipsoid in tissue with similar FA (Westin et al., 2002). A recent study of high school football players found that linear anisotropy was the most correlated DTI metric with head impact data taken from the players’ helmets (Davenport et al., 2014), suggesting that this metric may have increased sensitivity to the types of subtle axonal changes that are relevant for mild injury. In the current study, the combination of decreased linear and increased planar anisotropy in damaged white matter tracts may arise for several reasons, including axonal morphology changes, cell infiltration, and more complicated architectural changes observed following injury in the network of inflammatory cells surrounding particular parts of injured tissue such as the astrocytic network (Roth et al., 2014) or the robust and functionally significant morphologic changes undergone by microglia in response to injury (Ziebell et al., 2015).

While the brachium of the superior colliculus demonstrated many of the same histologic features as the optic tract including ample neuroinflammation and axonal disruption, DTI metrics were not as similarly altered as for the optic tract. AD in this region was not altered, but WL anisotropy was decreased, which may suggest greater sensitivity of the linear anisotropy measure compared with AD. Another explanation for the difference in DTI metrics in this region is that partial volume effects of this very thin structure reduce the sensitivity of the imaging metrics.

CHIMERA is primarily a white matter injury; however, DTI values were abnormal within the hippocampus, suggesting that CHIMERA may affect this vulnerable region as well as diffuse white matter tracts. DTI difference maps showed decreased FA in both hippocampi of the CHIMERA-injured samples, and group analysis found a decrease in FA, as well as a slight decrease in WL and WP. This DTI profile is distinct from changes in the white matter for which reduced FA was accompanied by changes in AD. Histology in this region showed an increase in APP immunoreactivity, which is indicative of potentially subtle axonal damage to the hippocampus. The alterations in DTI metrics and qualitative histology is indicative of potentially subtle damage to the hippocampus caused by CHIMERA injury. The damage caused to the hippocampus is not as copious as the damage seen in white matter tracts throughout the CHIMERA mouse brain yet is still detectable via DTI metrics.

Several studies have performed Pearson’s correlation analysis or similar statistics to determine the linear relationship between DTI metrics and histology or behavioral measures in studies of neurologic disorders. While this is intuitively attractive, several aspects of this type of analysis precluded the use of such an approach in this study. The study would require a much larger sample of controls and injured brains, to accurately investigate how neurophysiology correlates with DTI metrics in healthy tissue, and then to investigate how TBI neuropathology potentially alters these relationships. Correlation analysis could be useful if enough data were available to determine with high statistical certainty the correlation of metrics in each group and compare correlates in injured tissue to correlates in healthy tissue. Due to our experimental design, i.e., very high-quality MRI and detailed analysis of histology in a small number of animals, this approach would not be feasible here. The inferences made in this study between DTI parameters and histologic features are intended to further elucidate the multiple components of TBI neuropathology that DTI is sensitive to, not to characterize the reproducibility of the CHIMERA lesion in a population of animals. Instead of correlation analysis, the random forests analysis approach was implemented to determine the classification potential of each type of parameter collected in this study along with the importance of each ROI selected to successfully predict TBI status.

Perhaps the greatest remaining challenge for the effective use of DTI tools in brain research is the gap in understanding of the neurobiological correlates of DTI measures. Often, general associations between metrics are cited for example, FA being associated with “white matter integrity.” These may be misleading as numerous and distinct biological changes could result in the exact same change to DTI values. For example, a decrease in FA may arise from demyelination, axonal loss, crossing fibers, or infiltrating cells, each having a clearly different impact on the brain despite an identical FA value. Complete characterization of the multiple cellular and molecular components contributing to DTI metrics would require a very large battery of histology markers. Additionally, neuropathology will affect the way a DTI metric behaves in a particular region, especially in a complex neuropathology with multiple secondary injury mechanisms, as is the case in TBI. A study like the one described would require a large sample size and the measurement of multiple parameters. This type of analysis is beyond the scope of the current study; however, within the current study, we propose using a machine-learning algorithm that can handle a multiparametric dataset and show that it can easily extract important parameters for classification free of user bias.

### Classification of injury via DTI and histologic abnormalities

The question of which metrics are most affected by TBI and therefore most informative of TBI neuropathology was investigated. Classification of sham versus CHIMERA-injured brains via different DTI and histology metrics was examined via random forests analysis. When all DTI metrics were considered together, DTI was able to classify the majority of sham and CHIMERA samples. When all ROIs were considered for classification, one sham sample was misclassified and one CHIMERA sample was misclassified. When all histology markers were considered together, the same sham sample was misclassified by the histology yet the one CHIMERA-injured sample was correctly classified. DTI metrics were equivalent to histology markers in classifying sham samples. Histology markers, however, more accurately classified injured samples when compared to DTI metric classification.

We additionally ran random forests with a subset of DTI metrics as input variables (1) T and FA, (2) AD and RD, and (3) T, WL, WP (Table 6). Each of these trials had similar classification accuracy as when using all DTI parameters, which suggests that the selection of particular DTI metrics does not greatly change the information about injury that is conveyed by DTI in this model. There was one sham sample consistently classified as a CHIMERA-injured brain in all of the tests (all histology, all DTI, and all subsets of DTI). While this misclassification may arise for several reasons, it is likely related to the specimen preparation. It is important to note that both histology and DTI were sensitive to the differences in this sample (Tables 5, 6).

When looking at the top three best regions for classification, the algorithm primarily selected data from the ROIs identified as injured by group analyses of DTI metrics and histology markers (optic tract, brachium, hippocampus). The two regions selected as most important for classification in the models, the optic tract and brachium of the superior colliculus, were abundant in silver staining and showed reduced FA on the difference maps. Random forests has shown that it is capable of selecting injured ROIs in a manner unaffected by potential operator-induced bias. The data also shows that random forests can identify metrics that classify injury with 100% accuracy in an injury group which includes different injury severities (0.5J and 0.65J). This tool could prove clinically useful given that the TBI diagnosis results in a heterogeneous population consisting of several injury severities with no useful prognostic tools for separating the population. Other studies have shown the utility of random forests in identifying ROIs with DTI abnormalities in temporal lobe epilepsy (Chiang et al., 2016) and multiple sclerosis patients (Kacar et al., 2011). This study highlights how machine-learning algorithms may assist in the hunt for neuroimaging as well as other biomarkers of TBI in future studies.

This study was intentionally designed to obtain and compare comprehensive information within the same biological specimen including high-quality imaging and multiple histologic stains. Due to this design, the study is limited to having a small sample size (Sham, *n* = 5; CHIMERA, *n* = 5), which affects the statistical power for characterization of the average group outcomes for CHIMERA injury. This also limits the generalizability of the study results. The data presented are intended to introduce a classification approach that is novel to this type of study as well as a comprehensive understanding of the types of neurobiological changes that underlie diffusion metric. The question regarding neurobiological correlates of DTI metrics is a very important point of discussion (Budde et al., 2011; Harris et al., 2016). DTI is quickly proving to be a useful neuroimaging technique for a variety of neuropathologies (Chen et al., 2008; Adam et al., 2015; Melah et al., 2015; Loane et al., 2016; Nagae et al., 2016; Sener et al., 2016; Yang et al., 2017). However, the neurobiology underlying DTI abnormalities is still poorly understood. Other studies have addressed this question by correlating a particular histologic measure to a DTI metric, which can be an informative approach, but must acknowledge the contribution of other overlapping changes in brain tissue and be careful to not associate changes in a DTI metric with a singular cellular alteration. Rather, damage to brain tissue causes a number of different but often simultaneous cellular processes including microglia infiltration and proliferation, astrocyte swelling, Na-K pump breakdown inducing disruption of concentration gradients, excitotoxic neuronal death, and breakdown of myelin sheath compaction. Likewise, the current study only investigated a single time point after injury (7 d), which would not capture the complex array of cellular and molecular alterations that are changing over time. These dynamic alterations would affect DTI metric values to different extents, changing the relationship between histopathology and diffusion imaging at different times. All these cellular and molecular mechanisms contribute to changes in the water diffusion within a voxel, making it unlikely that a one-to-one correspondence will explain the relationship between histologic features and DTI metric changes. Instead of explaining DTI changes with neurohistology, DTI metrics should be viewed as an additional tool to help explain neuropathology, potentially with its own biological relevance. DTI has the added benefit of being a clinically available tool. CHIMERA initiates secondary injury, which is similar to human neuropathology after TBI and can be detected via a translatable tool. Using a more accurate neuropathological TBI injury model and a translatable tool to study that model, may improve understanding of the neurobiology of clinical TBI and may lead to more effective therapeutics.

### Conclusions

TBI is a highly prevalent neurologic condition yet lacks predictive diagnostics and effective therapeutics. Using TBI animal models that accurately replicate human TBI and focus on clinically translatable outcomes may aid in the search for biomarkers and therapeutics. A recently developed TBI injury model, CHIMERA, is reproducible and mimics DAI, a common injury pathology across clinical cases. This study comprehensively investigated the relationship between DTI and histologic metrics in CHIMERA-injured mice. DTI metrics were able to accurately capture DAI in numerous white matter tracts and in some gray matter regions in the CHIMERA model. The same regions showing DTI abnormalities also showed changes in histologic markers of injury. Remarkably, DTI metric abnormalities were colocalized with abnormalities in various histology markers indicating that multiple cellular mechanisms initiated by TBI may be responsible for the observed changes in DTI metrics. A machine-learning algorithm, random forests, was able to accurately detect regions identified as injured and classify injured samples using only DTI metrics. This study helps further elucidate the relevance of CHIMERA as a model of DAI and highlights the importance of DTI as a translational outcome of TBI. Further studies using carefully acquired radiologic-histopathological measurements in experimental TBI animal models, over multiple time points would elucidate translational methods with neurobiological meaning for TBI.

## Data accessibility

All data in this study will be made available on request from the corresponding author (M.H.).

## Synthesis

Reviewing Editor: Maiken Nedergaard, University of Rochester Medical Center Decisions are customarily a result of the Reviewing Editor and the peer reviewers coming together and discussing their recommendations until a consensus is reached. When revisions are invited, a fact-based synthesis statement explaining their decision and outlining what is needed to prepare a revision will be listed below. The following reviewer(s) agreed to reveal their identity: Selva Baltan, L Turtzo.

Reviewer #1

Mild traumatic brain injury is an epidemic of brain damage and a major challenge for scientists. Diffusion tensor imaging (DTI) is a newer imaging modality and provide improved edema and axonal injury detection. Closed-head Impact Model of Engineered Rotational Acceleration (CHIMERA) is a novel surgery-free model of traumatic brain injury that fully integrates biomechanical, behavioral and neuropathological analysis. This manuscript provides data on the ability of DTI to evaluate quantitative MRI markers of traumatic axonal injury in CHIMERA mouse model. Using ex vivo DTI maps together with histological analysis, pathology in specific white matter areas are identified. They concluded that reduced Fractional Anisotropy (FA) correlated with axonal damage and robust gliosis. Interestingly to avoid potential user bias, a machine learning algorithm detected injured regions with the similar degree of accuracy. The study confirms superiority of DTI over other MRI imaging modalities in detecting white matter injury using a novel mouse model and an unbiased algorithm.

High quality images, extensive statistical methods to quantify multiparametric data are provided. The manuscript is written clearly and with a fluent style. Some parts of the text is very technical which may suggest that a more suitable journal for imaging can be considered. The results mostly confirm literature in the aspect that TBI causes diffuse axonal damage. However the model and quantification methods provide a new approach. On the other hand there is no treatment group or continuum for acute, chronic/late injury changes or correlation with biomarkers such as S100B, NSE or tau. There are multiple aspects of the study to advance although the authors readily admit that these are preliminary studies. Nevertheless precision and timeliness of an accurate diagnosis is critical and necessary for diagnosis and medical treatment of TBI patients.

Specific Concerns are :

1) Table is helpful but can be combined with a Figure.

2) Figure 1- please label above the groups of the images their specific staining/modality etc such as column 1 is silver staining etc.

3) Apply the similar approach for Figure 2 - row 1 is silver staining for instance.

4) Table 3 is a challenge for a scientist who is not familiar with advanced statistics. For instance p = 0.00 and t8 values- please explain better. Does t8 value over 4 is the cut off for statistical significance when p value is adjusted? One is expecting that FA would have the most significant values while the table does not confer that. Somehow Table 4 display more expected results upon reading the text.

5) Figure 4 - APP labeling looks like increased in the CHIMERA (G) optic nerve - at least there is a streak that is darker - perhaps not uniform in other sections as statistics infer.

6) Figure 6- are statistics missing?

7) Figure 7 - label the images properly with letters and staining.

8) What is the fate of oligodendrocytes in the CHIMERA model ? If MBP labeling is more or less but axonal injury is evident with NF labeling that show axonal head and bulb formation - do oligodendrocytes stay viable?

Reviewer #2

This manuscript advances the field of preclinical TBI research by directly investigating relationships between ex vivo MRI findings with the underlying histopathology in the CHIMERA mouse model. They also demonstrate feasibility of using a machine learning based approach to classify sham versus injured animals based on the observed data. These results may help better understand the significance of similar findings on human in vivo MRI, if validated by further investigations.

The visual abstract would be even more informative if the authors A) add a text label indicating that these are all 7 days post-injury and b) make their arrows in the first two columns consistent with the location magnified in the third column. The location in the top row is more midline in the magnified area than in the MRI and first histopathology slide. The middle row is consistent. From the curvature the bottom row's magnified area appears to be from the right hemisphere of the image flagged in the first two columns, not the left.

This is a well-written manuscript summarizing a solidly designed investigation of the relationship between ex vivo MRI findings and histopathology in the CHIMERA mouse model of TBI. While similar studies have been done in other TBI models, this study is important for its better characterization of this potentially more clinically relevant preclinical model. The authors also demonstrate feasibility of applying a machine based learning approach to classify injured versus non-injured brain, in an attempt to limit bias. As a pilot investigation, the sample size is small and confined to one sex at a single endpoint (7 days post-injury), but the authors indicate their intention to take those factors into account in future studies. The figures and tables are illustrative and well-presented. The authors also include a detailed discussion of the limitations of their study, as well as acknowledging that DTI changes reflect a plethora of histopathological changes.

Two minor suggestions for the authors:

1) For your figures, I would suggest only marking those differences that remained significant after the multiple comparisons correction. That will limit confusion on the part of the reader, and the full details in the associated tables will clearly indicate which differences were statistically significant prior to the multiple comparison correction.

2) This study reflects analysis at a single time point (7 days post-injury, which would be considered to be a subacute time point). A point should be made in the discussion that the relationship between DTI findings and histopathology at other time points may be different, given the complexity of different findings associated both at the time point they investigated, as well as known changes at other time points. This study is a good start, but more comprehensive analysis will require looking both at more acute and more chronic time points to see the relationships longitudinally.

---------------------------------------------

Synthesis of Reviews:

The two reviewers liked the submission but had several critical comments that need to be addressed. The manuscript will be re-reviewed before a final decision is made.
